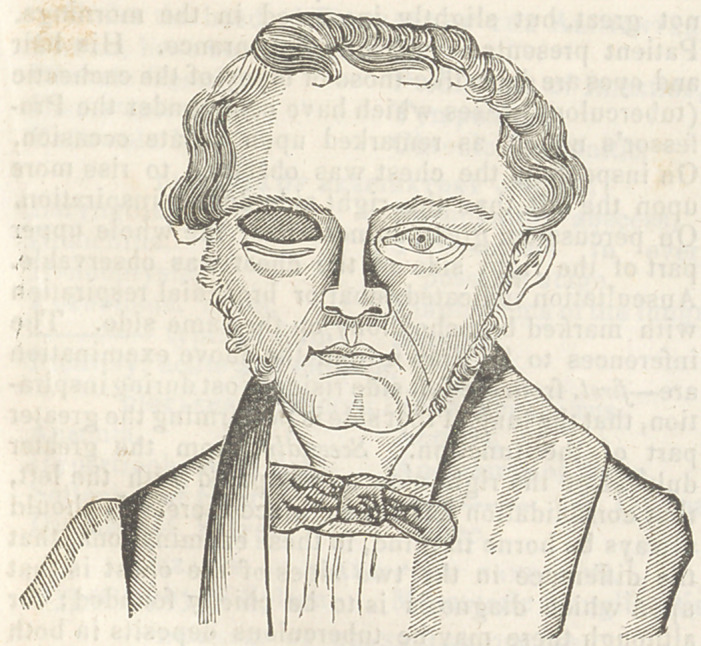# Operation for the Cure of Blephoroptosis

**Published:** 1844-05-04

**Authors:** J. Pancoast

**Affiliations:** Professor of Anatomy in Jefferson Medical College


### THE MEDICAL EXAMINER,

### And Record of Medical Science.

Vol. VII.] PHILADELPHIA, SATURDAY, MAY 4, 1844. [No. 9.

### OPERATION FOR THE CURE OF BLEPHOROPTOSIS.

BY J. PANCOAST, M. D.

Professor of Anatomy in Jefferson Medical College.

When this affection depends upon a complete paralysis of the levator muscle of the upper eye lid—the single agent by which the eye, is, in common language, opened—the lid hangs immovable before the ball, so as to completely obstruct vision. Fortunately this affection is rare, and limited usually to the organ of one side. It may merely constitute a part of a more general paralytic affection, depend solely upon a weakened power of contraction in the levator muscle, or exist merely as a symptom of hysteria, or intestinal irritation. The treatment of symptomatic palsy of the lid, is to be addressed chiefly to the primordial affection, and may in case the local lesion of the muscle, shows a tendency to become persistent, be singularly aided by the use of electro-puncture.

In cases of permanent palsy of the muscle, there is no prospect of relief afforded except by an operation. If the fall of the lid is only partial, the shortening of the lid by the removal of an elliptical portion of its integuments will sometimes suffice. But in case the ptosis is complete, and especially if there is reason to fear that there is a congenital deficiency of the muscle, the ingenious process of Hunt, of Manchester, by which the occipito-frontalis muscle is made to subserve the place of the levatór, presents the only prospect of relief.

Th s operation which will be understood by reference to the accompanying cut, I performed, June 19, 1842, at the clinic of the Jefferson Medical College, in the case of a yonng man, affected with congenital palsy of the levator muscle.

I made immediately below the eyebrow a curvilinear incision corresponding with the direction of the upper wall of the orbit, and of a length nearly equal to the fissure between the lids. From the ends of this another incision was made convex in the opposite direction, or towards the free edge of the lid. The included integument was then removed by a rapid dissection. The subcutaneous structure was next excised to some extent near the upper line of incision, so as to expose the lower edge of the occipito-frontalis. The edges of the divided skin were then approximated by four points of interrupted suture, which removed the deformity at once by lifting up the pendant lid to its proper position. On the second day, the sutures were removed; union had taken place throughout by first intention. A few narrow strips of adhesive plaster, were however, applied to support the parts. On the 26th of June, the patient was again presented at the clinic. The object of the operation had been completely obtained, the superciliary edge of the occipito-frontalis muscle, had acquired through the medium of the cicatrix an attachment to the tarsal edge of the lid, so as to give the patient complete voluntary control over the movements of the lid, which has remained permanent up to the present time.

## Figures and Tables

**Figure f1:**